# BIS-mediated STAT3 stabilization regulates glioblastoma stem cell-like phenotypes

**DOI:** 10.18632/oncotarget.9039

**Published:** 2016-04-27

**Authors:** Chang-Nim Im, Hye Hyeon Yun, Byunghoo Song, Dong-Ye Youn, Mei Nu Cui, Hong Sug Kim, Gyeong Sin Park, Jeong-Hwa Lee

**Affiliations:** ^1^ Department of Biochemistry, College of Medicine, The Catholic University of Korea, Seoul, Korea; ^2^ Institute for Aging and Metabolic Diseases, College of Medicine, The Catholic University of Korea, Seoul, Korea; ^3^ Cancer Evolution Research Center, College of Medicine, The Catholic University of Korea, Seoul, Korea; ^4^ Cancer Research Institute, College of Medicine, The Catholic University of Korea, Seoul, Korea; ^5^ NGS Clinical Department, Macrogen Inc., Seoul, Korea; ^6^ Department of Hospital Pathology, College of Medicine, The Catholic University of Korea, Seoul, Korea

**Keywords:** BIS, glioblastoma, CSCs, STAT3, ubiquitination

## Abstract

Glioblastoma stem cells (GSCs) are a subpopulation of highly tumorigenic and stem-like cells that are responsible for resistance to conventional therapy. Bcl-2-intreacting cell death suppressor (BIS; also known as BAG3) is an anti-apoptotic protein that is highly expressed in human cancers with various origins, including glioblastoma. In the present study, to investigate the role of BIS in GSC subpopulation, we examined the expression profile of BIS in A172 and U87-MG glioblastoma cell lines under specific *in vitro* culture conditions that enrich GSC-like cells in spheres. Both BIS mRNA and protein levels significantly increased under the sphere-forming condition as compared with standard culture conditions. BIS depletion resulted in notable decreases in sphere-forming activity and was accompanied with decreases in SOX-2 expression. The expression of STAT3, a master regulator of stemness, also decreased following BIS depletion concomitant with decreases in the nuclear levels of active phosphorylated STAT3, while ectopic STAT3 overexpression resulted in recovery of sphere-forming activity in BIS-knockdown glioblastoma cells. Additionally, immunoprecipitation and confocal microscopy revealed that BIS physically interacts with STAT3. Furthermore, BIS depletion increased STAT3 ubiquitination, suggesting that BIS is necessary for STAT3 stabilization in GSC-like cells. BIS depletion also affected epithelial-to-mesenchymal transition-related genes as evidenced by decrease in SNAIL and MMP-2 expression and increase in E-cadherin expression in GSC-like cells. Our findings suggest that high levels of BIS expression might confer stem-cell-like properties on cancer cells through STAT3 stabilization, indicating that BIS is a potential target in cancer therapy.

## INTRODUCTION

Glioblastoma is the most aggressive brain tumor and displays resistance to standard therapies, such as radiotherapy and chemotherapy, leading to poor prognosis and survival rates (~13–15 months) [1]. Recent studies demonstrated that glioblastoma exhibits remarkable heterogeneity, including a small population of self-renewing glioblastoma stem cells (GSCs) [2, 3]. In addition to self-renewal activity, GSCs share similar properties of normal stem cells, such as multilineage differentiation to astrocytes, oligodendrocytes, or neurons, as well as expression of a variety of stemness-related genes, including SOX-2 and NESTIN [4–6]. GSCs also exhibit cancer stem-cell-like features, such as invasion, modulation of immune response, and high motility, which likely contribute to the highly infiltrative nature of glioblastoma. More importantly, the extreme resistance by glioblastoma to standard therapy leading to frequent recurrence was attributed to the therapeutic resistance of GSCs to genotoxic stress or radiation due to preferential activation of DNA damage response [7]. These significant features of GSCs, even after gold-standard treatments, sustain the ability of the remaining cells to evade treatment and re-initiate propagation and infiltration. Thus, it is critical to understand the intrinsic molecular nature of GSCs in order to develop therapeutic approaches that target and eliminate them to prevent GSC-driven tumor progression and recurrence, which could overcome the limitation of conventional therapies that target bulk tumors [2, 8].

Signal transducer and activator of transcription 3 (STAT3) is a transcription factor that regulates cell proliferation, differentiation, and survival in response to a variety of cytokines and growth factors [9]. Constitutive activation of STAT3 by phosphorylation has been reported in 70% of human cancers, including glioblastoma [10]. In addition to its important role in oncogenesis, STAT3 is also involved in modeling of cancer stem cells (CSCs) of various origins, including breast, lung, pancreas, and head and neck cancers [11–15], suggesting that STAT3 inhibition is an approach to enhancing CSC-targeted therapy in human cancers.

Bcl-2-intreacting cell death suppressor (BIS), also known as BAG3, was originally identified as a Bcl-2-interacting protein that enhanced the anti-apoptotic activity of Bcl-2 [16]. Accumulating reports subsequently demonstrated that BIS was overexpressed in human cancers of various origins [17, 18], and the degree of BIS expression correlated with the poor prognosis of some cancers, such as pancreatic, thyroid, and glioblastoma [19–21]. Numerous *in vitro* and *in vivo* animal studies have indicated that modulation of BIS expression conferred sensitization or protection of cancer cells to chemotherapeutic agents, indicating the possibility that BIS could be a potential therapeutic target [22–24]. In addition to its pro-survival function, BIS reduction by RNA interference in a tumor xenograft suppressed invasion and metastasis *in vivo*, suggesting that high levels of BIS are relevant to tumor invasion and metastasis [25]. Xiao et al. also demonstrated that BIS knockdown consistently inhibited metastasis, as well as reversal of the epithelial-to-mesenchymal transition (EMT) pathway, in hepatocellular carcinoma [26]. Additionally, Suzuki et al. reported that BIS interacted with MMP-2 to positively regulate invasion by ovarian carcinoma cells [27]. Although there are discrepancies regarding the effect of BIS on EMT and/or metastasis [28, 29], BIS appears to play a key role in EMT and metastasis process. Considering that acquisition of EMT properties is closely related to CSC stemness [30–32], it is probable that BIS is involved in the regulation of CSC features, which is supported by the high expression of BIS in more aggressive tumors. Little is known about the role of BIS in the regulation of stemness in CSCs either *in vitro* or *in vivo*. Previously, we established the specific culture conditions that enriched GSC-like cell expression of SOX-2 [33]. In the present study, using this *in vitro* condition to A172 and U87-MG glioblastoma cell lines we investigate the effect of BIS knockdown on the regulation of GSC-like properties. We observed that BIS depletion suppressed GSC-like phenotypes including sphere formation, expression of stemness or EMT-related genes, which was accompanied by increase in STAT3 ubiquitination.

## RESULTS

### BIS knockdown inhibits sphere-forming activity of glioblastoma *in vitro*

**Figure 1:**
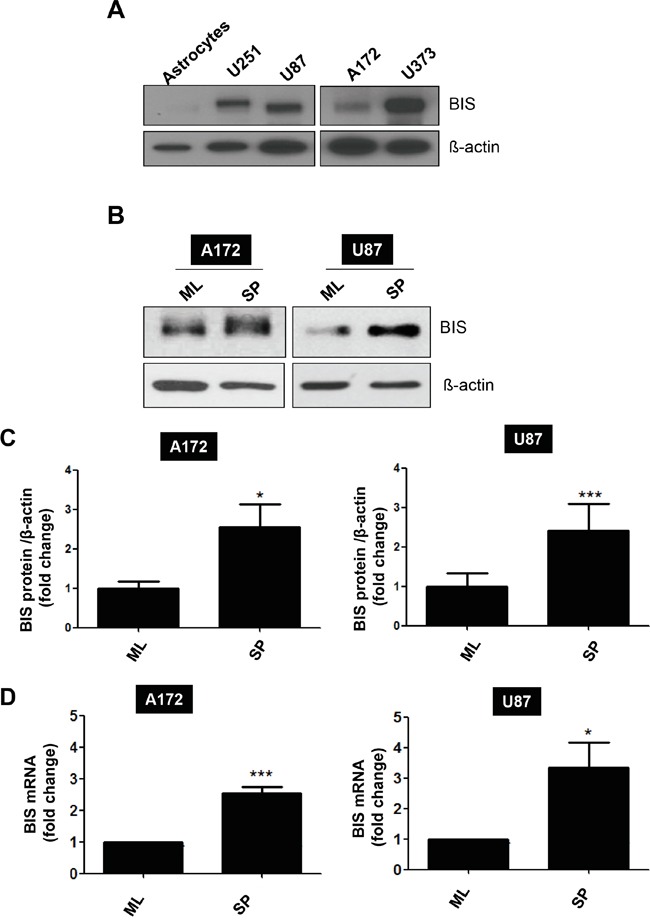
BIS induction under a specific culture condition that enriches GSC-like cells **A.** BIS levels were examined in various human glioblastoma cell lines, as well as normal astrocytes, by western blot assay. **B.** A172 or U87 cells were cultivated under standard monolayer (ML) or sphere-forming (SP) conditions for 72 h, followed by western blot assay to assess BIS expression levels. **C.** Relative expression levels were presented by densitometric analysis as BIS/β-actin ratio. Each value was expressed as mean ± SEM from three independent experiments **D.** BIS mRNA levels under each condition were evaluated by qRT-PCR. Each value was expressed as mean ± SEM from three independent experiments. *p < 0.05, ***p < 0.005 *vs*. ML

**Figure 2:**
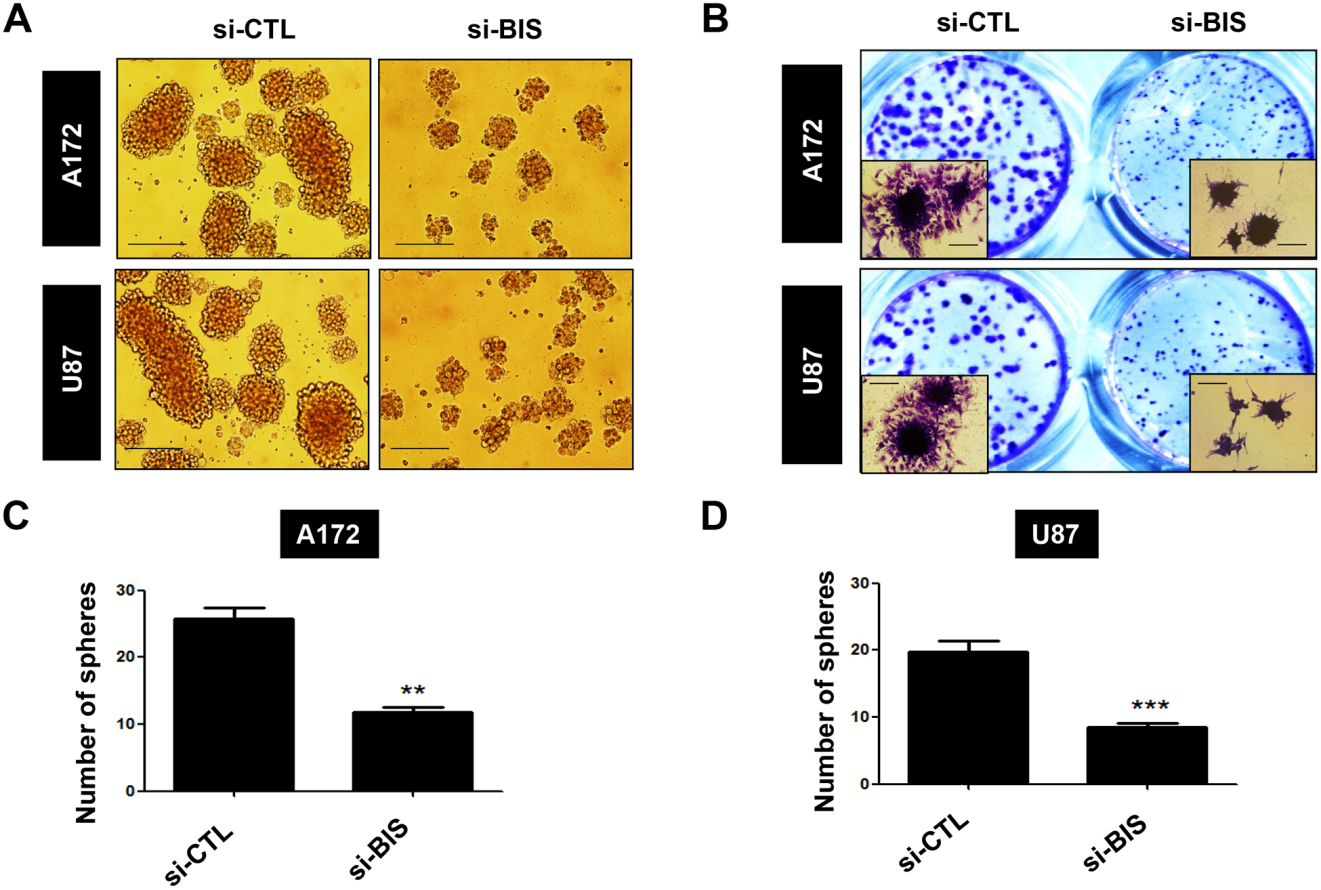
BIS knockdown inhibits sphere-forming activity A172 and U87 cells were transfected with 100 nM of control siRNA (si-CTL) or BIS-specific siRNA (si-BIS) for 48 h and subsequently cultured under SP conditions for 72 h. **A.** Morphology of spheres in A172 and U87 cells. Spheres were photographed by phase-contrast microscopy. Scale bar: 200 μm. **B.** Spheres were attached to standard culture plates for 6–8 h in media containing serum and stained with crystal violet solution (insert; magnification: 200×). The number of spheres was counted in **C.** A172 or **D.** U87 cells, and is represented as the mean ± SEM from three independent experiments.

**Figure 3:**
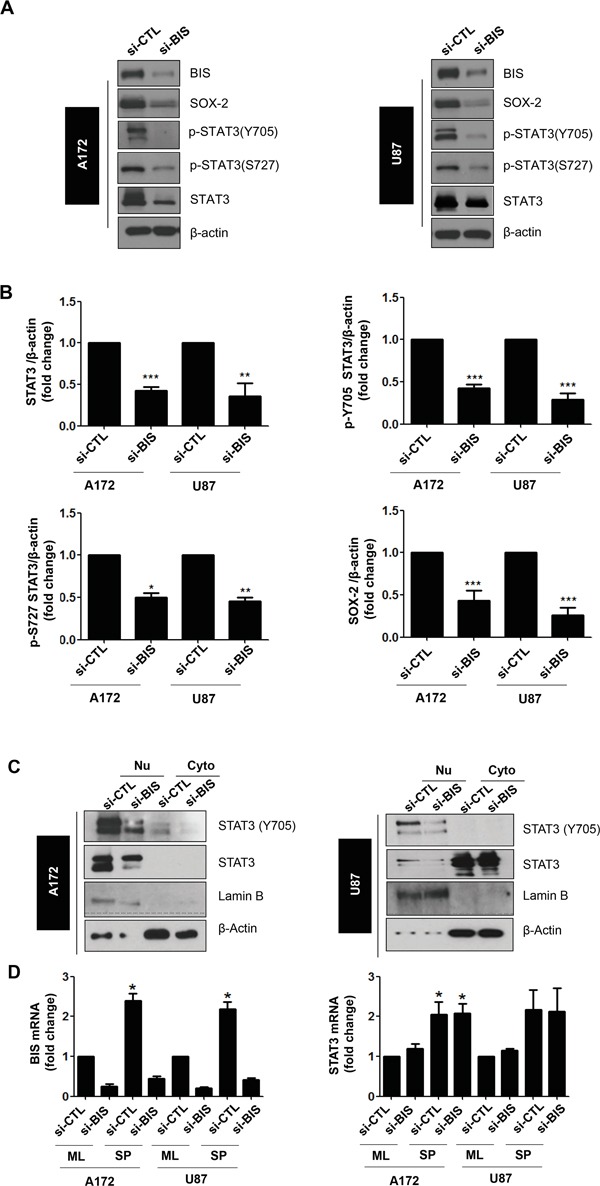
BIS knockdown decreased the total STAT3 protein, but not mRNA, levels in spheres of A172 and U87 cells **A.** The expression levels of indicated proteins were analyzed via western blot using specific antibodies. SOX-2 was shown as a stemness-related marker. **B.** Statistical analysis of total STAT3, p-Y705, p-S727, and SOX-2 protein levels in si-CTL or si-BIS-treated A172 and U87 spheres. Densitometric analysis was performed for protein levels of STAT3, p-Y705, p-S727 and SOX-2, from more than three independent western blots and the relative values of each protein to β-actin were represented as mean ± S.E. * *p* < 0.05, ** *p* < 0.01, *** *p* < 0.005 *vs.* si-CTL. **C.** Subcellular localization of phosphorylated STAT3 (p-STAT3) was demonstrated by western blot for p-STAT3 (Y705) after subcellular fractionation as described in Materials and Methods. Lamin B or β-actin was used as a marker for the nuclear (Nu) or cytoplasmic (Cyto) fraction, respectively. **D.** BIS and STAT3 mRNA levels were determined via qRT-PCR under standard culture (ML) or spheres-forming (SP) conditions after treatment with si-CTL or si-BIS. Values were expressed as the mean ± SEM from three independent experiments. * *p* < 0.05 *vs.* si-CTL in ML.

It was previously reported that BIS is highly expressed in samples from glioblastoma patients by immunohistochemical detection [21], which was corroborated by BIS mRNA expression in non-tumor and tumor samples from glioblastoma patients in a Repository for Molecular Brain Neoplasia Data (REMBRANDT) data set (Figure S1). Consistent with these findings, we also observed that BIS expression was higher in all glioblastoma cell lines tested as compared with normal astrocytes (Figure 1A). We then examined whether the expression profile of BIS was altered under the specific culture conditions for sphere formation, which enriches CSC-like cells expressing SOX-2 [33, 34]. Western blot assay shows that BIS expression levels increased under sphere-forming conditions (designated as SP) relative to standard monolayer culture conditions (designated as ML) in both A172 and U87-MG (designated as U87) glioblastoma cells (Figure 1B and 1C). Quantitative real-time PCR (qRT-PCR) analysis revealed that BIS mRNA levels increased by ~2.5- and 3-fold under sphere-forming conditions in A172 and U87 cells, respectively, indicating transcriptional activation of BIS expression might be concomitant with the enrichment process for GSC cells (Figure 1D). To elucidate the significance of increased BIS expression under sphere-forming conditions, we examined whether modulation of BIS expression affected sphere-forming activity, which was shown to be a representative property of GSCs [34]. A172 and U87 cells were transfected with small interfering RNA (siRNA) for BIS (si-BIS) or control siRNA (si-CTL) for 48 h, followed by incubation under sphere-forming conditions for a subsequent 72 h. As shown in Figure 2A, the size and the number of spheres notably decreased in si-BIS-treated cells as compared to si-CTL-treated cells in both A172 and U87 cells. The quantitation of colony numbers after attachment to normal plates revealed that BIS depletion reduced sphere-forming activity by ~50% relative to that of the control group (Figure 2B–2D). Additionally, BIS knockdown attenuated induction of the representative stemness marker SOX-2 in spheres (Figure 3A). Thus, BIS induction under sphere-forming conditions might be an essential prerequisite for expression of GSC-like properties.

### BIS knockdown inhibits STAT3 activity in glioblastoma cells under sphere-forming conditions

Since constitutive activation of the STAT3 pathway has been suggested as a critical requirement for the maintenance of cancer cell stemness [35], we examined the effect of BIS depletion on the STAT3 expression and nuclear translocation under sphere-forming conditions. We performed western blot assays for phosphorylated STAT3 (p-STAT3), which is the active form of STAT3 as a transcription factor, using specific antibodies targeting p-S727 and p-Y705 sites. In the spheres of A172 and U87 cells treated with si-BIS, p-STAT3 levels associated with both sites decreased relative to control cells (Figure 3A). We also noticed that total STAT3 levels were significantly lower in spheres from BIS-depleted A172 and U87 cells. Densitometry analysis exhibited similar profile in the decrease in total STAT3 as well as p-STAT3, indicating that the decreases in total STAT3 levels are mainly attributable to the reduction in p-STAT3 levels in BIS-knockdown A172 and U87 cells (Figure 3B). Accordingly, translocation of total STAT3 and p-Y705 STAT3 into the nucleus was inhibited by BIS depletion in the spheres of both A172 and U87 cells (Figure 3C). In re-attached cells following single cell dissociation from the spheres, the STAT3 intensity in the nucleus was obviously decreased by BIS depletion both in A172 and U87 cells (Figure S2). Notably, the appearance of STAT3 was more abundant in the cytoplasm of U87 cells as compared to A172 cells, which was a similar pattern to that observed following subcellular fractionation (Figures S2 and 3B). To determine whether the decrease in total STAT3 levels following BIS depletion was due to decreased STAT3 mRNA levels, we measured STAT3 mRNA levels under monolayer and sphere forming conditions by qRT-PCR. As shown in Figure 3D, we observed that STAT3 mRNA levels were elevated in the spheres of both A172 and U87 cells, which were not affected by BIS depletion. These results suggested that BIS depletion regulates STAT3 expression at post-transcriptional levels under sphere-forming conditions, resulting in decreased STAT3 phosphorylation and subcellular localization.

### Interaction of BIS and STAT3 regulates STAT3 ubiquitination

**Figure 4:**
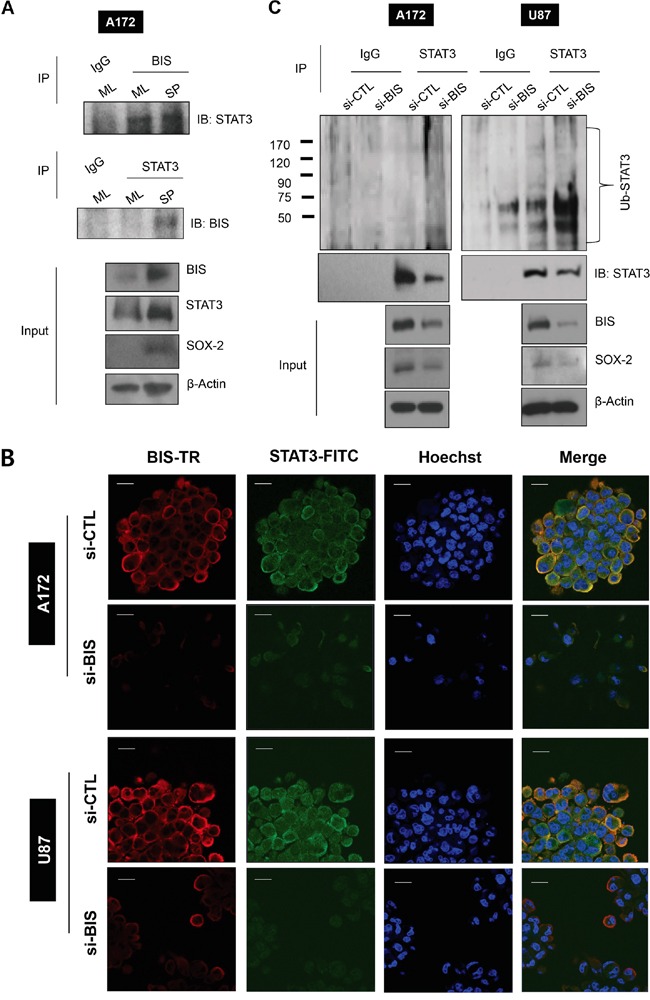
BIS interacts with STAT3 and BIS knockdown increases STAT3 ubiquitination **A.** Interaction between BIS and STAT3 was demonstrated by co-immunoprecipitation (IP) for endogenous BIS and STAT3 under ML or SP conditions. The cell lysates were immunoprecipitated with indicated antibody or normal immunoglobulin G (IgG), and then subjected to immunoblotting against STAT3 or BIS as indicated. The expression levels of BIS, STAT3, and SOX-2 were examined in the input lysates and compared with those of β-actin. **B.** BIS was co-stained along with STAT3 in spheres of A172 and U87 cells and observed by confocal microscopy. Hoechst 33342 was utilized for nuclear staining. Scale bar: 20 μm. **C.** STAT3 ubiquitination in spheres was analyzed by immunoprecipitation with the STAT3 antibody, followed by western blot with anti-ubiquitin (Ub) or STAT3.

Previously, we observed that BIS formed a complex with STAT3 in A172 cells under normal monolayer conditions [36]. Here, to investigate whether BIS and STAT3 interaction is involved in the induction of GSC-like phenotypes, we performed immunoprecipitation with a BIS polyclonal antibody or a STAT3 monoclonal antibody using the lysates from monolayer cells or spheres of A172. The immunoprecipitated complex was subsequently analyzed by western blot with a STAT3 or BIS antibody, respectively. As shown in Figure 4A, BIS protein formed an immune complex with STAT3 under both monolayer and sphere-forming conditions. Confocal microscopy confirmed that STAT3 co-localized with BIS in the spheres of A172 and U87 cells as shown by the merge of immunoreactive signals for each protein, and that BIS depletion decreased STAT3 immunoreactivity in the spheres of A172 and U87 cells (Figure 4B). In addition to *in vitro* glioblastoma cells, we also observed co-localization of BIS and STAT3 in human glioblastoma tissue (Figure S3). We then investigated whether decreases in BIS-depletion–mediated STAT3 were due to the accelerated degradation of STAT3 through the ubiquitin-proteasome pathway. Figure 4C shows that STAT3 ubiquitination increased following BIS depletion, which was concomitant with decreased STAT3 and SOX-2 levels. These data indicated that BIS stabilized STAT3, thereby protecting it from proteasome-mediated degradation.

### Ectopic STAT3 overexpression reverses the decrease in sphere-forming activity in BIS-depleted glioblastoma cells

**Figure 5:**
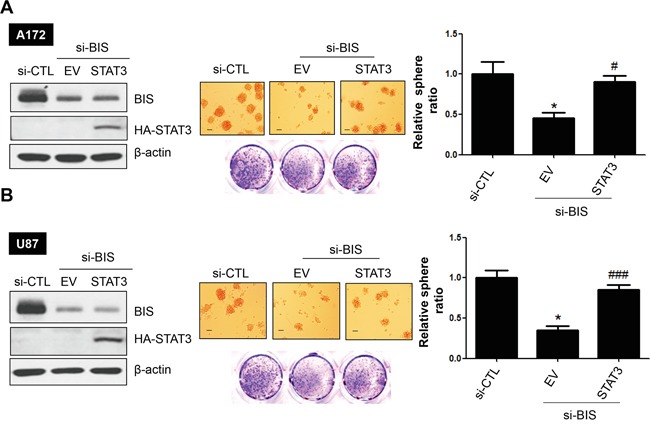
STAT3 overexpression reverses sphere-forming activity in BIS-knockdown glioblastoma cells Effects of STAT3 ectopic expression on sphere-forming activity in **A.** A172 and **B.** U87 cells treated with si-BIS. Hemagglutinin (HA)-tagged STAT3 plasmid was transiently overexpressed prior to si-RNA treatment. Western blots (left panel) and sphere-forming assays (middle panel) were performed. Spheres were counted and the relative ratio of sphere number to that of si-CTL was provided as the mean ± SEM from three independent experiments (right panel). Scale bar: 100 μm. * *p* < 0.05 *vs.* si-CTL. ### *p* < 0.005 *vs.* si-BIS.

To determine whether BIS-mediated regulation of STAT3 is essential to maintenance of GSC characteristics, we examined the effect of STAT3 overexpression on sphere-forming activity in BIS-depleted cells. A172 or U87 cells were transiently transfected with hemagglutinin (HA)-tagged STAT3 or an empty vector overnight, followed by treatment with si-BIS or si-CTL for a subsequent 48 h. After the cells were cultivated under sphere-forming conditions for 72 h, western blot analysis was performed for BIS or HA-STAT3 in order to verify the expression of each protein. As shown in Figure 5, sphere numbers were recovered by ectopic STAT3 expression in BIS-depleted A172 and U87 cells. In addition, SOX-2 expression was partially rescued by ectopic expression of STAT3 (from 37% to 69 % compared to the value in si-CTL –treated A172 cells, data not shown). Therefore, these results indicated that BIS-mediated stabilization of STAT3 represented a core element associated with the sustainment of a GSC phenotype.

### Effect of BIS depletion on the expression of stemness- and EMT-related genes in glioblastoma cells

**Figure 6:**
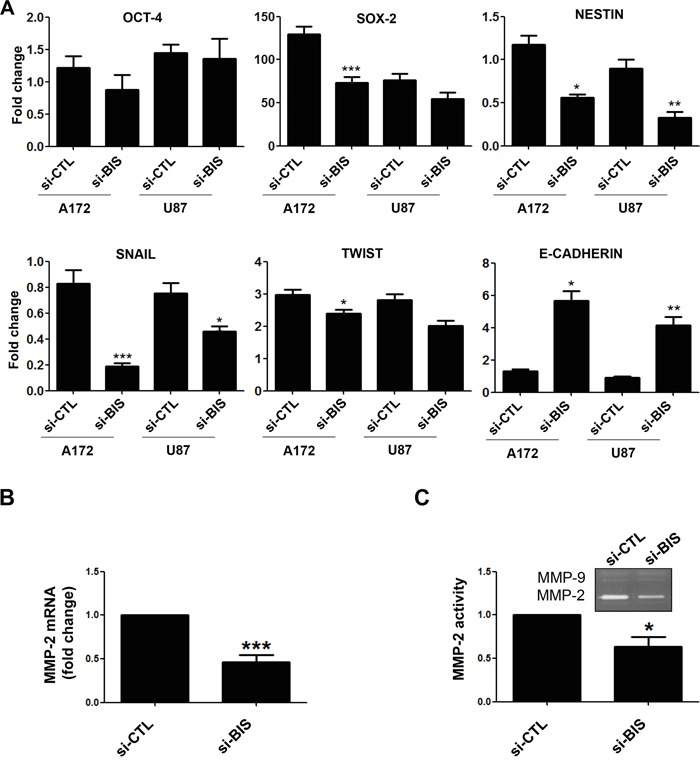
Alterations in the expression of stemness- or EMT-related genes by BIS depletion **A.** qRT-PCR was performed to determine the expression of stemness-related genes (OCT-4, SOX-2, and NESTIN) and EMT-related genes (SNAIL, TWIST, and E-CADHERIN) in the spheres of A172 or U87 cells treated with si-CTL or si-BIS. The expression of each gene after normalization to β-ACTIN in siCTL-treated cells in normal ML condition was designated as 1.0. The relative value was expressed as the mean ± SEM from three independent experiments. **B.** MMP-2 mRNA levels in the spheres from A172 cells were also determined by qRT-PCR. **C.** MMP-2 activity was demonstrated by zymography using supernatant from sphere-culture medium as described in Materials & Methods section followed by densitometric analysis. Representative zymography results are shown. Each value was expressed as the mean ± SEM from three independent experiments. * *p* < 0.05. ** *p* < 0.05. *** *p* < 0.005 *vs.* si-CTL.

CSCs display a high potential for EMT or vice versa [32, 37], and STAT3 was reported to activate the EMT phenotype by modulating the expression of EMT-related transcription factors, including TWIST, SNAIL, and ZEB1 [38, 39]. Therefore, to determine whether BIS is involved in EMT-related activity in glioblastoma cells, we examined the effect of BIS depletion on the expression of the principal epithelial and mesenchymal markers under sphere-forming conditions. Analysis of qRT-PCR revealed that BIS depletion reduced expression levels of the representative stemness-related genes SOX-2 and NESTIN in the spheres as compared with those observed in the control group (Figure 6A). Among EMT markers, SNAIL was significantly downregulated by BIS depletion, as was TWIST induction, though to a lesser extent than that observed with SNAIL. However, levels of E-cadherin, an epithelial marker, were notably higher in BIS-depleted cells, indicating that BIS depletion retarded the EMT process though regulation of the expression of several genes. Since BIS enhances the activities of MMP-2 [26, 27] and MMP-9 [40], which are representative STAT3 targets [41], we measured the mRNA levels of these genes. As shown in Figure 6B, MMP-2 mRNA levels significantly decreased in si-BIS-treated A172 spheres, but MMP-9 levels remained unchanged (data not shown). Consistently, MMP-2 activity, but not MMP-9, was inhibited by BIS depletion as determined by gelatin zymography using supernatants from culture media associated with A172 spheres (Figure 6C). Since targeting BIS alters the expression patterns of several genes associated with CSC and EMT properties, we hypothesized that BIS knockdown altered comprehensive expression profiles associated with STAT3 and EMT-related pathways. To test this hypothesis, we performed microarray analysis using Affymetrix arrays to assess the overall mRNA expression patterns in si-BIS-treated spheres as compared to si-CTL-treated spheres. As shown in Figure S4A, heat-map analysis revealed distinctly different patterns of gene expression in si-CTL-treated spheres as compared to the si-BIS-treated group. Among the differentially expressed genes, we selected CDKN1A, also known as p21, from the group exhibiting increased expression levels, and SOX-4 from the group exhibiting decreased expression levels, as representatives of the STAT3- and EMT-related pathways, respectively. RT-PCR (Table S1) verified that CDKN1A expression increased by up to 2.5-fold, while SOX-4 expression decreased by 0.5-fold, confirming that both mRNA levels were significantly suppressed by BIS depletion during the process of acquiring GSC-like phenotypes (Figure S4B and S4C). These data supported BIS contributing to the induction or maintenance of GSC-like phenotypes, possibly through modulation of the expression of many unidentified genes, though further study is required for confirmation.

## DISCUSSION

BIS is an anti-apoptotic protein that is highly expressed in various tumors, including glioblastoma. The significant inhibitory effects of BIS depletion on tumorigenesis in several mouse models, as well as on the invasive properties of cancer cells, imply the association of BIS with CSC functions. However, there are few studies on the relationship of BIS in the regulation of CSC-like properties. In the present study, we described, to our knowledge, for the first time, the role of BIS in a GSC subpopulation based on the following aspects: 1) BIS expression significantly increased under sphere-forming culture conditions as compared to monolayer conditions; 2) BIS depletion resulted in reduced stemness, as evidenced by decreases in sphere-forming activity and the expression of stemness-related genes, such as SOX-2 and NESTIN; 3) BIS knockdown decreased STAT3 levels, while STAT3 overexpression rescued sphere-forming activity; and 4) BIS depletion negatively affected EMT-related genes, such as SNAIL and MMP-2, displaying an association between stemness and EMT progression. Our results suggested that the high levels of BIS expression related to aggressive glioblastoma phenotypes contribute to the maintenance of GSC subpopulations. Even though BIS depletion reduced the SOX-2 levels, BIS depletion was not accompanied by the decrease in CD133 or CD44, which has been known to increase in several types of stem cells [5, 42] (data not shown). Consistent with our results, the recent reports raised questions in the application of CD133 as a reliable marker for cancer stemness [43, 44]. Thus, the essential role for CD133 or CD44 in the acquisition or maintenance of GSC properties remains to be clarified by further studies, especially in relation with BIS expression.

As an essential molecular mechanism through which BIS regulates GSC properties, we demonstrated that BIS depletion was linked to decreased STAT3 stability, and subsequently, nuclear translocation of active p-STAT3 under sphere-forming conditions. Considering that STAT3 physically interacts with BIS, and that BIS depletion enhanced STAT3 ubiquitination, the interaction of BIS and STAT3 might protect STAT3 from ubiquitin-proteasome-mediated degradation. Thus, the induction of BIS under sphere-forming conditions might be driven by necessity to stabilize STAT3, which is a major regulator of stemness phenotypes. A series of recent reports demonstrated that constitutive activation of STAT3 in glioblastoma, as well as concomitant activation of various upstream signals involving epidermal growth factor receptor, specific methyltransferase, or PI3 kinase, is regarded as an important regulator of GSC stemness [45–48]. Inhibition of upstream signals, as well as STAT3 using specific inhibitors or siRNA, significantly suppressed GSC sphere-formation, self-renewal activity, and tumor progression [45–47, 49], suggesting that inhibition of STAT3 signaling may be a therapeutic target for clinical applications associated with glioblastoma. Thus, our results suggested that BIS depletion is an effective strategy for directly targeting STAT3 to accelerate its degradation. Recently, two groups independently reported the successful delivery of BIS siRNA to glioblastoma or squamous cell carcinoma models using peptide-like polymers or gold-nanorod-siRNA nanoplexes [50, 51]. These findings indicated that the increased efficacy of BIS-siRNA delivery techniques might advance the therapeutic application of BIS targeted to suppress tumor progression by inhibiting the anti-apoptotic effect of BIS, as well as STAT3-mediated CSC progression.

Protein homeostasis, referred to as proteostasis, is finely regulated by the balance of protein synthesis and degradation to meet cellular requirements under various conditions [52]. Proteins are degraded through two major pathways: the ubiquitin-proteasome pathway and the autophagy-lysosome pathway [53]. Accumulating evidence suggests that BIS is involved in protein quality control by promoting the autophagy-lysosome pathway in order to clear aggregation-prone proteins in cooperation with HSC70/HSP70, HSPB8 and p62 [54, 55]. On the other hands, BAG1, another BAG family protein has been suggested to direct HSP70-bound ubiquitinated client proteins to the proteasome through ubiquitin-like domain [56]. Thus depletion of BIS, which disrupts the balance between BIS/BAG1 expression ratio, might shift the direction of major protein degradation pathway toward ubiquitin-proteasome machinery as previously suggested [57, 58]. In addition, BIS has been known to interact with HSP70 [59], which recruits ubiquitin ligase and directs the client proteins to proteasome. Through its interaction with HSP70 or client protein directly, BIS has been shown to inhibit the proteasomal delivery of several HSP70 client proteins including Bcl-xL, BRAFF, Mcl-1 and IKKγ, resulting in the stabilization of these HSP70-client proteins [60–63]. Thus, it is possible that, by forming complex with STAT3 and/or by sequestering HSP70, BIS might confer STAT3 protecting from HSP70-driven ubiquitination and subsequent proteasome-mediated degradation. On the other hands, even though BIS has no enzymatic domain, the BIS interactome and bioinformatics analysis revealed that BIS interacts with several proteins functioning in the proteasome-ubiquitination process [64]. Thus, it is also probable that BIS directly modulates the activity of proteasome machinery, in addition to protects the client proteins.

Recently, we observed that BIS was induced during myogenic differentiation, and that BIS depletion increased ubiquitination and subsequent degradation of myosin heavy chain (MHC), resulting in significant atrophy of myofibrils [65]. These results indicated that the molecular association of BIS with MHC or STAT3 is essential for maintenance of myofibril mass or sphere-forming properties, which confer specific characteristics on muscles or CSCs, respectively. Through binding with multiple proteins to subsequently confer stabilization, BIS contributes to the preservation of various cellular functions, including pro-survival activity, muscle contraction, and cancer cell stemness. Therefore, disruption of the interaction of BIS with specific proteins may result in disturbances of cellular functions dependent upon the nature of the target proteins. This could be applied as a therapeutic strategy associated with various pathological conditions; however, investigation of the signaling pathways that determine the binding preference for BIS among its multiple partners, and identification of the functional domains related to BIS interactions, should proceed in order to develop small molecules capable of interfering with these activities.

In summary, the present study demonstrated that BIS depletion suppressed the stemness phenotypes associated with glioblastoma cells as evidenced by decreases in the sphere-forming activity and the expression of stemness- and EMT-related genes, likely following STAT3 destabilization. Further studies using patient-derived glioblastoma cells to analyze the characteristics of subpopulations in regard to the degree of BIS expression will clarify the significance of BIS as a therapeutic target and a reliable stemness marker.

## MATERIALS AND METHODS

### Cell culture

All human glioblastoma (A172, U87-MG, U251, U373) cell lines were purchased from American Type Culture Collection (ATCC, Manassas, VA, USA) and maintained in Dulbecco's Modified Eagle Medium (DMEM) containing 10% heat-inactivated fetal bovine serum (FBS; Hyclone, Logan, UT, USA), 100 U/mL penicillin, and 100 mg/mL streptomycin at 37°C in 5% CO2 atmosphere. Primary human brain astrocyte cells were provided by Cell Systems (Kirkland, WA, USA). BIS knockdown was performed by transfection of 100 nM of specific siRNA targeting BIS with G-fectin (Genolution Pharmaceuticals, Seoul, Korea) according to manufacturer instruction. The si-CTL (5′-CCUACGCCACCAAUUUCGU-3′) and si-BIS (5′-AAGGUUCAGACCAUCUUGGAA-3′) were purchased from Bioneer (Daejeon, Korea). STAT3 overexpression was performed as described previously [36].

### Sphere-formation assay

For the sphere-forming assay, a single-cell suspension following trypsinization was cultured in B27-supplemented DMEM/F12 (Cellgro, Manassas, VA, USA) with epidermal growth factor and basic fibroblast growth factor (10 ng/mL each: R&D Systems, Minneapolis, MN, USA) without serum on ultralow attachment plates at a density of 1 × 10^5^ cells/mL as described previously [33]. After 3–5 days, spheres were attached to standard culture plates in media containing 5% FBS stained with crystal violet solution (Sigma-Aldrich, St. Louis, MO, USA). For morphological examination, pictures were taken under the inverted microscope. Spheres above 50 μm in diameter were counted and expressed as mean ± SEM.

### Western blot

Cells were lysed with lysis buffer [150 mM NaCl, 1% NP-40, 0.5% sodium deoxycholate, 0.1% SDS, 50 mM Tris–HCl (pH 8.0)] with protease inhibitor (Roche Diagnostics, Mannheim, Germany) on ice for 30 min. Equal amounts of protein were separated by 10% SDS-PAGE and transferred to nitrocellulose membranes (GE Healthcare Life Sciences, Buckinghamshire, UK). The membranes were incubated for 1 h with 5% dry skim milk in Tris-buffered saline and Tween 20 (20 mM Tris, 137 mM NaCl, 0.1% Tween 20) and incubated with antibodies against BIS (1: 5000; [16]), p-STAT3 (1:1000; Y705, S727; Cell Signaling, Danvers, MA, USA), STAT3 (1:1000, Cell Signaling), SOX-2 (1: 500; Santa Cruz Biotechnology, Dallas, CA, USA), or β-actin (1:5000, Sigma-Aldrich). After incubation with horseradish peroxidase-conjugated secondary immunoglobulin G (IgG, 1: 5000; Santa Cruz Biotechnology), the immunoreactive bands were visualized by an enhanced chemiluminescence substrate (Thermo Fisher Scientific, Waltham, MA, USA). Quantification of the intensities of each band was carried out using Image J software (National Institutes of Health, Bethesda, MD, USA).

### Subcellular fractionation

Cells were incubated in buffer A [10 mM HEPES, 1.5 mM MgCl_2_, 10 mM KCl, 0.5 mM dithiothreitol (DTT), and 0.05% NP40 (pH 7.9)] for 10 min on ice. After centrifugation at 3000 rpm for 10 min, the supernatant was utilized as a cytosolic fraction. The pellet was suspended in buffer B [5 mM HEPES, 1.5 mM MgCl_2_, 0.2 mM EDTA, 0.5 mM DTT, 300 mM NaCl, and 26% glycerol (pH 7.9)], homogenized 20 times with a plastic pestle homogenizer, and incubated on ice for 30 min. After centrifugation at 13,200 rpm for 30 min, the supernatant was used as a nuclear fraction. Lamin B (Santa Cruz Biotechnology) was utilized as a nuclear-marker protein.

### qRT-PCR

Total RNA was isolated using AccuZol (Bioneer), and the first strand of cDNA was synthesized by reverse transcription using a ReverTra Ace qPCR kit (Toyobo, Osaka, Japan). qRT-PCR was performed to validate the expression levels using SYBR Premix Ex TaqTM (TaKaRa Bio, Shiga, Japan) with specific primers (Table 1) in an Applied Biosystems 7300 machine (Applied Biosystems, Carlsbad, CA, USA). The relative values for specific mRNA were calculated after normalization to the Ct value from β-actin in the same sample using the ddCt method.

**Table 1:** Primers for qRT-PCR

Gene	Sequence (5′-3′)
BIS	Forward: 5′-GGAATTCGCATGAGCGCCGCCACCCACTCG-3′
	Reverse: 5′-CTCGAGCTACGGTGCTGCTGGGTTACCAGG-3′
STAT3	Forward: 5′-AAGTTTATCTGTGTGACACC-3′
	Reverse: 5′-CTTCACCATTATTTCCAAAC-3′
OCT-4	Forward: 5′-CAGCGACTATGCACAACGAGA-3′
	Reverse: 5′-GCCCAGAGTGGTGACGGA-3′
SOX-2	Forward: 5′-TACCTCTTCCTCCCACTCCA-3′
	Reverse: 5′-ACTCTCCTCTTTTGCACCCC-3′
NESTIN	Forward: 5′-CGGTGGCTCCAAGACTTCC-3′
	Reverse: 5′-GGCACAGGTGTCTCAAGGGTA-3′
SNAIL	Forward: 5′-CCTCCCTGTCAGATGAGGAC-3′
	Reverse: 5′-CCAGGCTGAGGTATTCCTTG-3′
TWIST	Forward: 5′-GGAGTCCGCAGTCTTACGAG-3′
	Reverse: 5′-TCTGGAGGACCTGGTAGAGG-3′
E-CADHERIN	Reverse: 5′-TGCCCAGAAAATGAAAAAGG-3′
	Reverse: 5′-GTGTATGTGGCAATGCGTTC-3′
MMP-2	Forward: 5′-GCTGGAGACAAATTCTGGAG-3′
	Reverse: 5′-AGCTTCAGGTAATAGGCACC-3′
β-ACTIN	Forward: 5′-AGTACTCCGTGTGGATCGGC-3′
	Reverse: 5′-GCTGATCCACATCTGCTGGA-3′

STAT3, Signal transducer and activator of transcription 3; OCT-4, octamer-binding transcription factor 4; SOX-2, SRY (sex determining region Y)-box-2; SNAIL, SNAI1; MMP-2, matrix metalloprotease-2

### Confocal microscopy

Spheres or cells from spheres treated with trypsin were slightly attached on poly-L-ornithine-treated plates were fixed for 10 min with 3.7% formaldehyde in PBS and permeabilized for 10 min at room temperature with 0.2% Triton X-100. The cells were blocked for 1 h with 1% bovine serum albumin in PBS at room temperature. A specific primary antibody was added to the cells, which were incubated overnight at 4°C. The cells were washed in phosphate-buffered saline (PBS) and incubated for 1 h at 4°C with fluorescent Texas Red or fluorescein isothiocyanate-conjugated anti-rabbit or -mouse IgG (Santa Cruz Biotechnology). After washing, the cells were stained with 1.0 μg/mL Hoechst 33342 (Sigma-Aldrich) for 10 min at room temperature. Fluorescent images were acquired via confocal microscopy (Carl Zeiss, Oberkochen, Germany).

### Immunoprecipitation

Cell cultures or spheres were lysed with buffer [(10 mM HEPES, 0.4% NP-40, 142.5 mM KCl, 5 mM MgCl_2_, 1 mM EGTA (pH 8.0)] with protease inhibitor (Roche Diagnostics) on ice for 30 min. An equal amount of each protein lysate was incubated with the indicated antibodies, normal rabbit IgG, or normal mouse IgG (Santa Cruz Biotechnology) for 4 h at 4°C, followed by incubation with 20 μL of protein A magnetic beads (Millipore, Billerica, MA, USA) for 16 h at 4°C. The immune complexes were analyzed by western blot with the indicated antibodies. Total protein lysates were also subjected to western blot analysis with the indicated antibodies.

### Gelatin zymography

Gelatin zymography was performed using supernatants from sphere media cultivated in GBM on UP as previously described [66]. Briefly, conditioned media was separated by10% SDS-PAGE containing 0.2% gelatin. The gel was renatured with 2.5% Triton X-100 buffer for 1 h and incubated with developing solution (50 mM Tris, pH 7.5, 10 mM CaCl_2_) for 18–20 h at 37°C. The gel was then stained with 0.5% Coomassie Brilliant Blue (Sigma-Aldrich) in 30% methanol and 10% glacial acetic acid and destained with the same solution without dye.

### Statistics

The Student t-test was used to compare the differences between two groups. Each experiment was repeated at least three times. In all analyses, p < 0.05 was taken to indicate statistical significance.

## SUPPLEMENTARY FIGURES AND TABLE


