# Supplementary material for: BIS-mediated STAT3 stabilization regulates glioblastoma stem cell-like phenotypes
Source: Oncotarget. 2016 Apr 27;7(23):35056–70. doi: 10.18632/oncotarget.9039 (PMC5085209; doi:10.18632/oncotarget.9039)
Supplement: Supplementary file 1 [file oncotarget-07-35056-s001.pdf]

SUPPLEMENTARY DATA

SUPPLEMENTARY MATERIALS AND METHODS

Fluorescent immunohistocytometry

Nervous system glioma tissue arrays were purchased from Abcam (Cambridge, UK). The sections were deparaffinized in xylene and rehydrated in graded ethanol. Prior to staining, antigen retrieval for 15 min using a cooker in 10 mM standard sodium citrate buffer (pH 6.0) was performed for all antibodies used in this study. Nonspecific binding sites were blocked with phosphate-buffered saline (PBS) containing 5% bovine serum albumin. Immunoreaction with the primary antibodies was performed overnight at 4°C, followed by incubation with the appropriate secondary antibodies conjugated with fluorescent dye for 2 h at room temperature. Nuclei were stained with Hoechst 33342.

Microarray analysis

Spheres cultivated in sphere forming condition were harvested after 3 days, and microarray analysis was performed using Affymetrix GeneChip human 2.0 ST arrays (Macrogen, Seoul, Korea). For quality control, RNA purity and integrity were evaluated by OD 260/280 ratio, and analyzed using an Agilent 2100 Bioanalyzer (Agilent Technologies, Santa Clara, CA, USA). The Affymetrix Whole Transcript Expression array process was executed according to manufacturer protocol (GeneChip Whole Transcript PLUS reagent Kit; Macrogen). cDNA was synthesized using the GeneChip WT Amplification kit (Macrogen) as described by the manufacturer. The sense cDNA was then fragmented and biotin-labeled with terminal deoxynucleotidyl transferase using the GeneChip WT Terminal labeling kit (Macrogen). Approximately 5.5 μg of labeled DNA target was hybridized to the Affymetrix GeneChip Human 2.0 ST Array at 45°C for 16 h. Hybridized arrays were washed and stained on a GeneChip Fluidics Station 450 and scanned on a GCS3000 Scanner (Affymetrix, Santa Clara, CA, USA). Signal values were computed using the Affymetrix GeneChip Command Console software. Raw data were extracted automatically using the Affymetrix data-extraction protocol using the software provided by Affymetrix GeneChip Command Console Software (Affymetrix). After importing the CEL files, the data were summarized and normalized using a robust multi-average (RMA) method implemented in the Affymetrix Expression Console Software (Affymetrix). We exported the result with gene-level RMA analysis and preformed differentially expressed gene (DEG) analysis. The comparative analysis between the test sample and the control sample was carried out using an Independent t test and fold change, wherein the null hypothesis was that no difference existed between groups. A false discovery rate was controlled for by adjusting the p value using the Benjamini-Hochberg algorithm. For DEG sets, hierarchical cluster analysis was performed using complete linkage and Euclidean distance as measures of similarity.

All Statistical tests and visualization of differentially expressed genes was conducted using the R statistical language v. 3.1.2. (www.r-project.org).

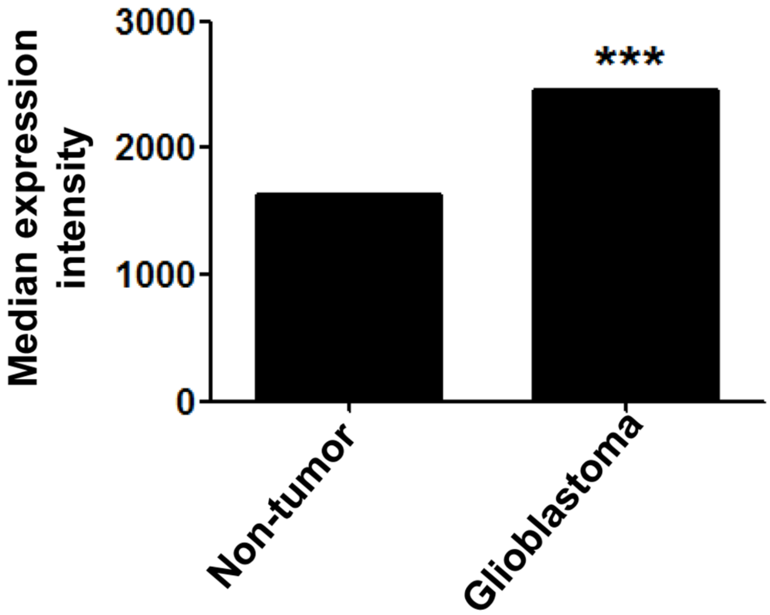

Supplementary Figure S1: Differential BIS expression in glioblastoma tissue. Median expression intensity of BIS (probe 217911) in the REMBRANDT dataset represents that BIS expression in glioblastoma tumor tissue is enhanced compare to that observed in non-tumor tissue. *** $p < 0.0005$.

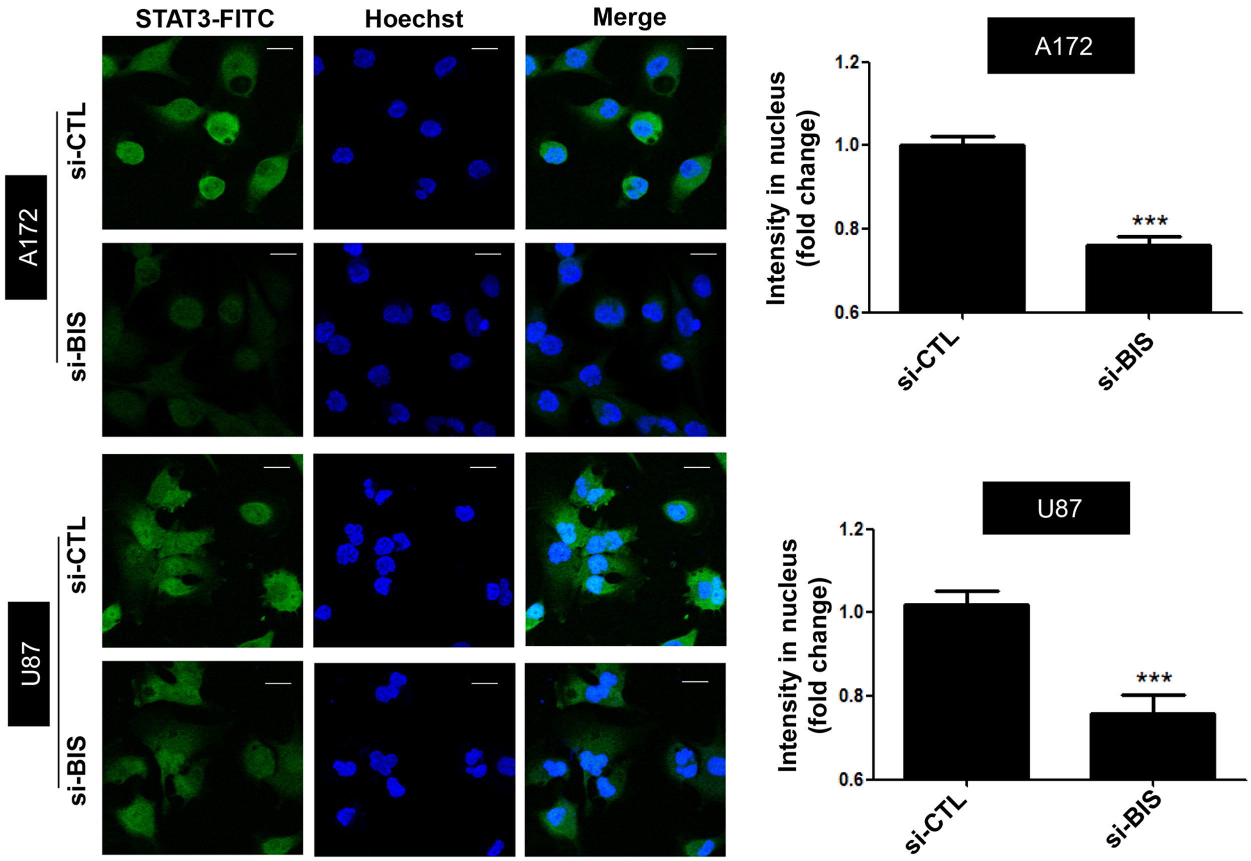

Supplementary Figure S2: Confocal microscopy in single cells. After transfection of si- BIS for 48 h, cells were seeded to ultralow attachment plates under SP conditions. After cultivation for 72 h, spheres were trypsinized and transferred to ornithine-coated slides for 12 h. Cells were fixed, treated with specific antibodies, and analyzed by confocal microscopy. Hoechst 33342 was utilized for nuclear staining. Scale bar: 20 µm (left panel). The quantification of STAT3 intensity in the nucleus of re-attached single cell from spheres of A172 or U87 cells. The mean value in si-CTL-treated cells was designated as 1.0 (right panel). *** $p < 0.005$ *vs*. si-CTL.

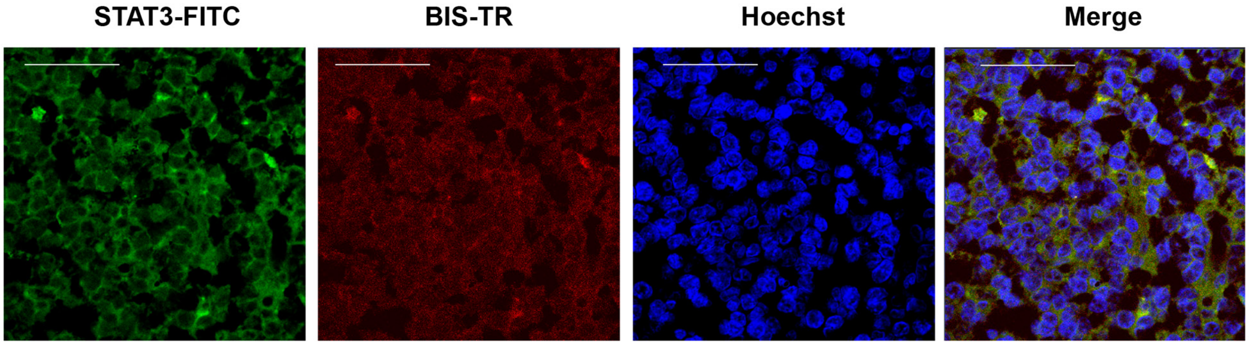

Supplementary Figure S3: STAT3 and BIS expression of in human glioblastoma tissue. Measurements were obtained by fluorescent immunohistochemistry. Scale bar: 50 µm.

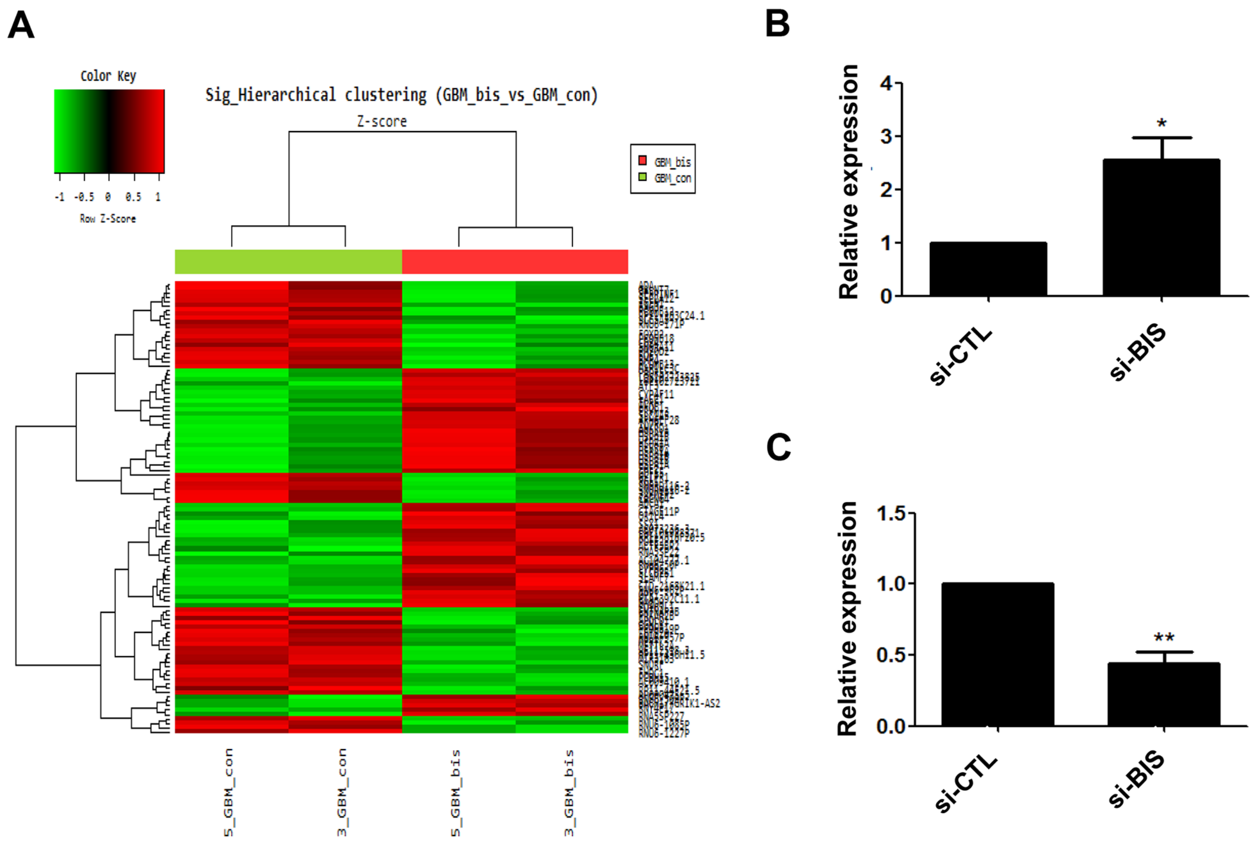

Supplementary Figure S4: Global expression patterns in A172 spheres. A. Heat-map analysis using microarray and qRT-PCR results for **B.** CDKN1A and **C.** SOX-4 expression among differentially expressed genes from three independent experiments. * $p < 0.05$ vs. si-CTL. ** $p < 0.01$ vs. si-CTL.

Supplementary Table S1: Primers for qRT-PCR

Gene	Sequence (5′-3′)
CDKN1A	Forward: 5′-GGAAGGGACACACAAGAAGAA-3′
	Reverse: 5′-TCCTTGTTCCGCTGCTAATC-3′
SOX-4	Forward: 5′-CTCTAGTTCTTGCACGCTCTTTA-3′
	Reverse: 5′-GTAGCTCAGGAAAGCGACATAG-3′
β-ACTIN	Forward: 5′-AGTACTCCGTGTGGATCGGC-3′
	Reverse: 5′-GCTGATCCACATCTGCTGGA-3′

SOX-4, SRY (sex determining region Y)-box-4; CDKN1A, cyclin-dependent kinase inhibitor 1A
